# The association between health expenditure, institutions, and economic growth in MENA countries

**DOI:** 10.34172/hpp.2022.12

**Published:** 2022-05-29

**Authors:** Bashir Umar Faruk, Mohammad Imdadul Haque, Mohammad Rumzi Tausif, Md Riyazuddin Khan

**Affiliations:** ^1^Department of Economics, Faculty of Management & Social Sciences, Federal University Gusau, Zamfara State, Nigeria; ^2^Department of Economics, Faculty of Social Science, Aligarh Muslim University, Aligarh, India; ^3^College of Business Administration, Prince Sattam Bin Abdulaziz University, Saudi Arabia; ^4^Department of Geography, Dr. Bhim Rao Ambedkar College, University of Delhi, New Delhi, India

**Keywords:** Health, Per capita income, Governance, Granger causality, Panel regression

## Abstract

**Background:** Expenditure on health is vital in the development of a country. Furthermore, the current COVID-19 pandemic emphasises the importance of health investments in maintaining a healthier economy worldwide. A substantial amount of empirical research on the relationship between health expenditure and economic growth yields conflicting results. The study intends to investigate the relationship between health spending and economic growth and institutions’ role in causing health spending to promote growth.

**Methods:** The study uses longitudinal data to examine the relationship between health spending and economic growth in seven MENA countries from 2000 to 2017. The study uses the Phillips Perron (PP) Fisher chi-square stationarity test, indicating that the data series is not stationary. Following this, we used the Pedroni test for cointegration, and the results show long-run relationships between the variables. Next, Granger causality determines the direction of causality. Finally, panel data methods of panel ordinary least squares (Panel OLS), fully modified OLS (FMOLS), and dynamic OLS (DLOS) supplement the findings.

**Results:** The Pedroni cointegration test (*P* value<0.0001) indicates that the variables have a long-run cointegrating relationship. On the other hand, the Granger causality test finds no causal relationships between health spending and economic growth. Furthermore, the panel data models show that expenditure on health does not directly contribute to higher economic growth in MENA countries.

**Conclusion:** The findings of this study indicate that health spending does not lead to increased economic growth; this could be due to poor institutional quality. However, for health spending to positively impact economic growth, these investments in health care must be supplemented by other factors, particularly institutions.

## Introduction

 Investment in health boosts labour productivity, which lowers the cost of lost human capital. Furthermore, expenditures in health result in lower mortality and morbidity, leading to a higher proportion of working-age people in the population and higher per capita incomes.^1^Healthy people are more energetic, enthusiastic, and productive, which is good for the country.^2^ Health impacts labour productivity since it enables intellectual and physical development.^3^ As a result, health spending is critical to enhancing the ‘human factor,’ which promotes economic growth via technical advancement and labour productivity.^4-7^

 Systemic problems beset the healthcare system in the Middle East and North Africa. Non-communicable disease rates are incredibly high in this region due to food and lifestyle changes and, government health spending has not grown significantly. These factors impact the quality and quantity of healthcare services available.^8^ The frequency of non-communicable and chronic diseases rises as the population of old aged people expands, along with the overall population.^9^ As a result, the significance of health spending in MENA economic growth merits additional research. The ongoing COVID-19 outbreak underscores the importance of examining healthcare systems.

 According to the World Health Organization, the health of a country’s population impacts its growth rate. It is estimated that a ten-year rise in life expectancy at birth will boost economic growth by 0.3% to 0.4%.^10^ Health investments, on the other hand, are insecure due to poor institutions, government failure, and a lack of transparency.^11^ Poor-quality institutions harm people’s health, which has a detrimental impact on economic growth. Although the importance of healthcare spending in economic growth is widely accepted; it depends on the country-specific quality of institutions as to whether further health investments will have a larger or smaller impact on the economic growth of the country.^12^

 Institutions and their functioning are critical for an economy’s general growth and understanding the health sector’s performance. Economic growth, social development, and total economic development are all influenced by the functioning of government institutions.^13^Regrettably, institutional governance in health care is still poorly understood.^14^ In developing countries, rising health spending is influenced by demand and supply and institutional factors.^15^ Health investments result in more substantial economic growth as institutions strengthen.^16^ When institutional quality interacts positively with health capital, it results in more effective resource allocation and economic growth.^17^

 There are also two competing opinions on the relationship between health spending and economic growth: the ‘health view’ and the ‘income view’. The health view proposes that income is a function of health. Because of improvements in health or an increase in health expenditure, there is an increase in income. At the same time, the other income view proposes that health is a function of income. Income is identified as the principal factor explaining the variances between countries regarding the share and level of healthcare expenditure. This mechanism of reverse causality claims that as income increases, people demand more and better health services. Past research on South Asian countries,^12^ African countries,^13^ sub-Saharan African (SSA) countries,^17^ and select countries in Europe, Middle East Africa and Asia,^18^ have experimentally tested these two ideas.

 This research aims to see how healthcare spending affects economic growth. One of the most critical measures of a country’s development is its healthcare system.^19^ Despite this, experts have only recently begun to pay attention to the importance of health capital.^20,21^ Also, insufficient attention has been paid to institutional quality in the relationship between economic growth and health spending. Furthermore, the scarcity of research in this area of the MENA region adds to the significance of this work. This study attempts to fill a knowledge gap revealed by the lack of research on the relationship between health expenditure and economic growth in MENA nations.

 Only a few studies have looked at healthcare spending in MENA nations and its relationship with economic growth.^22,23^ However, the role of the institution was overlooked in these investigations. The research intends to discover a long-term link between health spending and economic growth in MENA nations. The study’s originality incorporates institutions while controlling for life expectancy, household consumption, labour force, and trade indicators. The remainder of this study is divided into four sections: literature review, data and methods, results and discussion, and conclusion and suggestions.

###  Literature review

 Previous empirical researchers have found a long-run cointegration relationship between health spending and economic growth. Piabuo and Tieguhong^13^ for 12 African countries and Mehrara et al^24^for 13 MENA countries are two examples. Jamison et al^25^found health improvements attributed to 0.23% growth per year in a study of 53 countries. Rizvi^16^found that increasing health expenditures by 100% led to a 5% increase in economic growth when adjusted for the quality of government expenditures in a study of twenty Pacific and Southeast Asian developing countries. Sarpong et al^17^ found that a 1% increase in health expenditure led to a 0.20% increase in economic growth in another study of 35 SSA countries. Boussalem et al^26^found a cointegrating relationship between healthcare spending and economic growth in Algeria. In his study on Tunisia, Sahnoun^15^ found that a 1% increase in health spending increased economic growth by 0.43%.

 Some other studies investigated the causal relationships between healthcare expenditure and economic growth. Piabuo and Tieguhong^13^ found bi-directional causality between growth and health expenditure in countries with lower health expenditure, but only unilateral causality from growth to health expenditure in countries with higher health expenditure. Sarpong et al^17^ found a bidirectional relationship between economic growth and health spending. Sethi et al^12^found a bidirectional relationship between economic growth and health spending. The study also found a unidirectional causality between institutional quality and health expenditure. Boussalem et al^26^ found long-run causality between public health spending and economic growth in Algeria. Alhassan et al^27^ found a negligible negative impact of health spending on economic growth in Nigeria and unidirectional causality from health spending to economic growth.

 Lacheheb et al^22^found that health expenditure and education had a significant positive impact on economic growth in MENA countries. This implied that investments in health and education would boost these countries’ economic growth. Beraldo et al^4^ found that health and education expenditures contribute and compensated more than tax system distortions caused by increased welfare expenditures in another study conducted on 19 OECD countries from 1971 to 1998. In another survey of 92 countries, Silva et al^21^ discovered that increased health was positively associated to economic growth. Countries experiencing economic slowdowns or comparatively low growth rates benefitted more from health investments. Piabuo and Tieguhong^13^ found a significantly positive effect of health expenditure on economic growth in African countries, which was more significant in countries with comparatively higher allotted health expenditure.

 On the other hand, Awaworyi Churchill et al^28^ based their study on a meta-analysis of 306 estimates from 31 primary studies that report an adverse effect of government health spending on economic growth. The study explains the findings by claiming that health spending leads to inefficient allocation of public resources and crowding out factors. Moreover, when combined with distortionary taxation, it tends to alter saving decisions. This has a negative impact on growth because taxes are excessively high. Because of the crowding out of both productive private and public resources and tax distortions, increases in public health spending has a negative impact on economic growth. This occurrence is more likely in developed OECD and EU countries. Furthermore, the population in these developed countries are ageing, with chronic illnesses and multi-morbidities. These older people require extensive health care, but these investments in healthcare do not directly promote productivity because they are not contributing to the workforce. The same study also reports that other factors, such as the efficiency and quality of public health expenditure and poor mobility, also result in an inefficient impact of increased health expenditure and improvements in human capital.

 Furthermore, in their study on Tunisia, Ghorbel and Kalai^29^ found an inverse relationship between healthcare expenditure and economic growth, and causality tests showed that the two variables are not related. Mehrara and Musai^23^ found only a one-way effect from gross domestic product (GDP) to health spending and no causality running from health spending to economic growth. In his study on Saudi Arabia, Alhowaish^30^ found no cointegrating relationship between the variables. But the study reported unidirectional causality from economic growth to health spending. The study, however, found no evidence of a link between health spending and economic growth. Similarly, Nathaniel and Khan^31^ found that public health spending did not implicitly contribute to the quality of life in their research on Nigeria. The presence of contradictory results suggests that the relationship between health spending and economic growth has mixed results, necessitating further investigation.

## Materials and Methods

 The study examines seven MENA countries from 2000 to 2017. The sample countries namely, Saudi Arabia, Iran, Oman, Qatar, Tunisia, Cyprus, and Israel, are referred to as countries 1, 2, 3, 4, 5, 6, and 7 in the remainder of this paper. The study could not to include all the remaining MENA countries due to a lack of data. GDP per capita as a proxy for economic growth, health expenditure as a percentage of GDP, life expectancy (a proxy for health outcome), household consumption, labour force, trade as a percentage of GDP, and institutional factors are all considered in the study. GDP per capita has been used as a proxy for economic growth in empirical studies such as, Sethi et al,^12^ Sarpong et al,^17^ Mehrara et al,^24^ and Alhassan et al^27^ Institution is a simple average of the following World Bank governance indicators: “corruption control, government effectiveness, political stability and absence of violence/terrorism, regulatory quality, the rule of law, voice and accountability.” All variables, except for institutions, are in natural log form. All the data is taken from World Development Indicators and is analysed using E-views 9.0.

###  Empirical strategy

 This study used the same theoretical model as Piabuo and Tieguhong,^13^ emphasising the importance of human capital in economic growth. The model, in particular, has expressed a functional relationship between a critical component of human capital, that is, health expenditure and economic growth. The same echoes in the endogenous growth model. The functional relationship, on the other hand, has been modified to include institutions as follows:

 GDP_it_= α_i_+ β_i_HE_it_+γ_i_HC_it_+δ_i_LE_it_+ω_i_LF_it_+φ_i_TR_it_+ψ_i_INS_it_+∂_i_LHE*INS_it_+ε_it_

(1)

 where *i* is the individual country component, t stands for time component from 2000 to 2017, α is an intercept and β, γ, δ, ω, φ, ψ and ∂ are the coefficients and ε is the error term. GDP per capita represents economic growth, while HE stands for health expenditure per capita, HC for household consumption expenditure, LE for individual life expectancy at birth, LF for the proportion of the population that makes up the labour force, and TR for trade as a percentage of GDP, which is the sum of exports and imports of goods and services. LHE* INS denotes the interaction terms between health expenditure and institution. Incorporating trade is based on the assumption that a healthier society has higher labour productivity, producing more goods and services. Finally, INS stands for institutional excellence.

 The first step is to use the panel unit root test to determine whether a unit root exists in the data series. If the test results using either augmented Dickey-Fuller (ADF) or Phillips Perron (PP) Fisher-type turned out to be integrated of order one, and then Pedroni’s panel cointegration test will be used. To estimate the long-run relationship between the variables the study uses the absence of cointegration as its null hypothesis and the presence of cointegration as its alternate hypothesis. The following is the definition of the cointegration relationship:

 LGDP_it_= α_i_+λ_t_+ β_i_LHE_it_+γ_i_LHC_it_+δ_i_LLE_it_+ω_i_LLF_it_+φ_i_LTR_it_+ψ_i_INS_it_+∂LHE*INS_it_+ε_it_

(2)

 where α_i_ implies country effectsmeans a dummy for each country (except for one). So, the country specific fixed effect is modelled as a country-specific intercept which does not vary over time which captures the heterogeneity across countries and *λ*_t_ refers to trend effects that allow controlling for underlying observable and unobservable systematic differences between observed time units. *ε*_it_ is the estimated residual which shows the deviations from the long-run relationship. The Granger Causality test is used in this study to determine the direction of causality between health expenditure, economic growth and institution, and other control variables. Finally, the study uses panel ordinary least squares (Panel OLS), fully modified OLS (FMOLS), and dynamic OLS (DLOS) to estimate the relationship between the cointegrating variables specified in the model. The study uses the statistical software ‘E-views (version 9)’ for empirical analysis.

## Results

 An overview of the data (shown in Appendix 1) indicates that country 7 has the highest health expenditure per capita, followed by country 2, country 6, country 1, country 5, country 3, and country 4 over the study period (2000-2017). Countries have varying experiences with health expenditure per capita. Health expenditure in country 1 fluctuated between 4.21% and 5.23% over the years. It increased from 4.73% to 8.65% for country 2. It was 3.06% in 2000 for country 3 but was less than 3% from 2004 to 2013 and was 3.89% in 2017. Country 4 experienced a fluctuation between 2.00% and 2.60% over the years. Country 5 saw a continuous increase from 5.04% to 7.23%. Country 6 has seen a significant increase from 5.30% to 6.68%. It indicates an upward trend for country 7, increasing from 6.80% to 7.40% over the sample period.

 Over the study period (2000-2017), per capita GDP increased steadily in all listed MENA countries studied. Country 4 has the highest average GDP per capita, followed by country 7, country 6, country 3, country 2, country 5, and country 1. Country 3 received the highest institutional quality score, followed by country 4, country 6, and country 7. Three countries received negative scores. Country 7 has the lowest score, followed by country 1 and 2. The table on descriptive statistics contains additional information on the control variables of household consumption, labour force, and trade (Appendix 1).

 To assess the relationship between variables, the data should be checked for the presence of unit root, that is, stationarity. Panel unit roots tests are used in the study to see if there are any series of interests that are stationary or not. These tests have the null hypothesis that the series is non-stationary individually, against the alternative hypothesis that the series is stationary. The panel unit roots test used are Im, Pesaran and shin, ADF and PP Fisher-type tests, Levin, Lin & Chu t*, and Breitung t-stat,

Table 1 presents the results of panel unit root tests. The stationarity results based on the PP-Fisher chi-square, show that none of the data series is stationary. In other words, they are first-order integrated. Based on this finding, the study uses the Pedroni test to determine whether the variables have a long-run cointegration relationship or not.

**Table 1:** Panel unit root test results

**Test method**	**Level **	**First difference**	**Decision**
1. lm, Pesaran and Shin W-Stat	Intercept & Trend	Intercept & Trend	
	Test stat. (*P* value)	Test stat. (*P* value)	
			
4. Levin, Lin & Chu t*			
5. Breitung t-stat			
LGDP			
1	2.611 (0.996)	-4.425 ( < 0.000)	I(1)
2	5.190 (0.983)	43.561 ( < 0.000)	I(1)
3	3.860 (0.996)	67.181 ( < 0.000)	I(1)
4	0.329 (0.629)	-6.739 ( < 0.000)	I(1)
5	1.907 (0.972)	-2.488 (0.006)	I(1)
LHE			
1	1.056 (0.855)	-1.928 (0.027)	I(1)
2	7.376 (0.919)	24.645 (0.038)	I(1)
3	8.371 (0.869)	62.698 ( < 0.000)	I(1)
4	0.614 (0.731)	-0.528 (0.299)	I(2)
5	-0.884 (0.188)	-1.759 (0.039)	I(1)
LLE			
1	-22.133 ( < 0.000)	-11.134( < 0.000)	I(0)
2	101.961 ( < 0.000)	94.967( < 0.000)	I(0)
3	18.311(0.193)	35.781(0.001)	I(1)
4	-16.262 ( < 0.000)	-12.653( < 0.000)	I(0)
5	-2.337(0.010)	-0.868(0.193)	I(0)
LHC			
1	2.538 (0.994)	-3.374 ( < 0.000)	I(1)
2	3.854(0.996)	37.398 ( < 0.000)	I(1)
3	3.830 (0.996)	48.507 ( < 0.000)	I(1)
4	1.037(0.850)	-4.371 ( < 0.000)	I(1)
5	1.853(0.968)	-1.162 (0.123)	I(2)
LLF			
1	0.293 (0.615)	-0.415(0.339)	I(2)
2	14.715(0.398)	20.430(0.117)	I(2)
3	6.592(0.949)	33.565(0.002)	I(1)
4	-1.789(0.037)	-1.947(0.026)	I(0)
5	2.386(0.992)	0.599(0.725)	I(2)
LTR			
1	-0.571(0.284)	-2.768(0.003)	I(1)
2	17.063(0.253)	29.673(0.009)	I(1)
3	10.049(0.759)	60.385( < 0.000)	I(1)
4	-1.823(0.034)	-2.945(0.002)	I(0)
5	1.272(0.898)	-2.840(0.002)	I(1)
INST			
1	0.778 (0.782)	-5.594( < 0.000)	I(1)
2	9.193(0.819)	51.721( < 0.000)	I(1)
3	18.083(0.203)	89.801( < 0.000)	I(1)
4	0.259(0.602)	-5.982( < 0.000)	I(1)
5	0.092(0.537)	-3.160( < 0.000)	I(1)

Source: Researchers’ computation using E-views 9.0. Note: LGDP is logarithm value of gross domestic product per capita; LHE is logarithm value of health expenditure per capita; LHC is logarithm value of for Household Consumption expenditure; LLE is logarithm value of for individual life expectancy at birth; LLF is logarithm value of for the proportion of the population that makes up the labour force; LTR is logarithm value for trade as a percentage of GDP, which is the sum of exports and imports of goods and services.

 According to the cointegration result in Table 2, four of the seven within-dimension and between-dimension tests are significant under normal statistics at a 1% significance level. However, two of the four within-dimension tests are statistically significant underweighted statistics, one at a 1% significance level and the other at a 5% significance level. As a result, we reject the null hypothesis and conclude that cointegration between the variables exists.

**Table 2:** Panel cointegration test results

**Pedroni residual Cointegration test**		**Statistic (** ***P*** ** value)**	**Weighted statistic (** ***P*** ** value)**
Within-Dimension (Panel)	Panel v-statistic	1.3759 (0.0844)	-2.5771 (0.9950)
	Panel rho-statistic	3.1560 (0.9992)	3.3774 (0.9996)
	Panel PP-statistic	-16.0120 (0.0000)**	-2.8231 (0.0024)**
	Panel ADF-statistic	-4.0178 (0.0000)**	-2.1375 (0.0163)*
Between-Dimension (Group)	Group rho-statistic	4.4269 (1.0000)	
	Group PP-statistic	-10.6880 (0.0000)**	
	Group ADF-statistic	-5.0259 (0.0000)**	

PP, Phillips Perron; ADF, augmented Dickey-Fuller. *0.05 & ** < 0.01, under the null hypothesis of non-cointegration Source: Researchers’ computation using E-views 9.0.

 In other words, economic growth (GDP per capita), health expenditure, health outcome (life expectancy), household consumption expenditure, labour force, trade, and institutional quality all have a long-run cointegrating relationship. Long-run cointegrating relationships indicate that all variables are moving in the same direction, indicating that the variable’s trend remains consistent over a long period.

 The study also employs a pair-wise Granger causality test to determine the direction of causality between these variables. The null hypothesis asserts that no Granger causality exists between the variables. The test also determines whether the causality (if any) is unidirectional, bidirectional, or non-directional. The absence of causality indicates that previous values of one variable cannot explain the current values of the other variable.

 The findings show that GDP per capita has no causal relationship with other variables (Table 3). The study, in particular, discovers no link between economic growth and healthcare spending. However, labour force Granger causes health expenditure, but health expenditure Granger does not cause labour force. According to the Granger causality test, trade causes life expectancy, but life expectancy does not cause trade. Similarly, the causality between labour force participation and household consumption expenditure runs from labour force participation to household consumption, not the other way around.

**Table 3:** Granger causality test result

			**Observation**	**F-statistics**	***P*** ** value**
LHE	⇏	LGDP	112	0.59706	0.5523
LGDP	⇏	LHE	112	0.43767	0.6467
LLE	⇏	LGDP	112	0.35749	0.7003
LGDP	⇏	LLE	112	0.6582	0.5199
LHC	⇏	LGDP	112	1.34349	0.2953
LGDP	⇏	LHC	112	1.20999	0.3022
LLF	⇏	LGDP	112	1.74357	0.1798
LGDP	⇏	LLF	112	1.92686	0.1505
LTR	⇏	LGDP	112	0.36347	0.6961
LGDP	⇏	LTR	112	0.34461	0.7093
INS	⇏	LGDP	98	2.31004	0.1049
LGDP	⇏	INS	98	2.13358	0.1242
LLE	⇏	LHE	112	0.00584	0.9942
LHE	⇏	LLE	112	0.26777	0.7656
LHC	⇏	LHE	112	1.00208	0.3705
LHE	⇏	LHC	112	2.20907	0.1148
LLF	⇏	LHE	112	4.1493	0.0184^**^
LHE	⇏	LLF	112	0.66214	0.5178
LTR	⇏	LHE	112	0.20146	0.8178
LHE	⇏	LTR	112	0.23452	0.7914
INS	⇏	LHE	98	0.94923	0.3908
LHE	⇏	INS	98	2.3366	0.1023
LHC	⇏	LLE	112	0.01787	0.9823
LLE	⇏	LHC	112	1.1349	0.3253
LLF	⇏	LLE	112	0.33984	0.7126
LLE	⇏	LLF	112	2.03204	0.1361
LTR	⇏	LLE	112	2.66987	0.0739^*^
LLE	⇏	LTR	112	0.14512	0.8651
INS	⇏	LLE	98	1.74545	0.1802
LLE	⇏	INS	98	0.37084	0.6912
LLF	⇏	LHC	112	3.96216	0.0219^**^
LHC	⇏	LLF	112	2.15085	0.1214
LTR	⇏	LHC	112	1.54621	0.2178
LHC	⇏	LTR	112	4.92108	0.0090^***^
INS	⇏	LHC	98	2.84191	0.0634^*^
LHC	⇏	INS	98	0.09852	0.9063
LTR	⇏	LLF	112	2.2772	0.1075
LLF	⇏	LTR	112	2.19892	0.1159
INS	⇏	LLF	98	0.84196	0.4341
LLF	⇏	INS	98	1.13066	0.3272
INS	⇏	LTR	98	2.93999	0.0578^*^
LTR	⇏	INS	98	0.41714	0.6602

Null Hypothesis. (The sign ⇏ indicates ‘does not Granger cause’). Source: Researchers’ computation using E-views 9.0. Note: LGDP is logarithm value of Gross Domestic Product per capita; LHE is logarithm value of health expenditure per capita; LHC is logarithm value of for Household Consumption expenditure; LLE is logarithm value of for individual life expectancy at birth; LLF is logarithm value of for the proportion of the population that makes up the labour force; LTR is logarithm value for trade as a percentage of GDP, which is the sum of exports and imports of goods and services; LHE* INS denotes the interaction terms between logarithm value of health expenditure and institution and INS stands for institutional excellence.

 Furthermore, a unidirectional relationship exists between household consumption expenditure and trade and between household consumption and institutions. The causality runs from household consumption expenditure to trade, institutions to household consumption, and not the other way around. The outcome demonstrates a unidirectional relationship that runs from institutions to trade. All other relationships were found to be devoid of a causal link.

 Next, the study attempts to estimate the impact of the explanatory variables on GDP per capita using various panel data methods. The explanatory variables are health expenditure, labour force, household consumption expenditure, trade openness, life expectancy, institutions and an interaction term of health expenditure and institutions. The coefficients are supposed to have positive signs, while the thrust is on finding the impact of health expenditure and institutions on economic growth.

Table 4 shows that in all three models, namely Panel OLS, FMOLS, and DLOS, a significant negative relationship is observed between health expenditure (LHE) and GDP per capita on the one hand, and also between labour force (LLF) and GDP on the other hand (DOLS). This result demonstrates that a unit change in healthcare spending or labour force will decrease GDP per capita.

**Table 4:** Panel regression results

**GDP per capita (Dependent variable)**	**Panel OLS**	**FMOLS **	**DOLS**
	**Coefficient (** ***P*** ** value)**	**Coefficient (** ***P*** ** value)**	**Coefficient (** ***P *** **value)**
LHE	-1.516(0.000)**	-0.318(0.000)**	-0.375(0.000)**
LLE	8.990(0.000)**	-3.486(0.580)	-4.297(0.473)
LHC	0.921(0.000)**	1.012(0.000)**	1.035(0.000)**
LLF	-0.974(0.000)**	-0.481(0.000)**	-0.490(0.001)**
LTR	0.083(0.561)	0.448(0.000)**	0.429(0.000)**
INS	-0.099(0.002)**	0.043(0.095)	0.013(0.644)
LHE*INS	0.025(0.254)	-0.029(0.064)	-0.014(0.398)
C	-35.359(0.000)**	-	-
R-square	0.957	0.996	0.996
Adjusted R-square	0.954	0.995	0.995
Long-run variance		0.0038	0.0057

OLS, ordinary least squares; FMOLS, fully modified ordinary least squares; DOLS, dynamic ordinary least squares). ** denotes significant at 1% significant levels. Source: Researchers’ computation using E-views 9.0.

 However, in all three models, the relationship between household consumption expenditure (LHC) and GDP per capita is positive and statistically significant. Similarly, although trade (LTR) and GDP per capita have positive and significant relationships in FMOLS and DOLS, the relationship is statistically insignificant in Panel OLS. As household consumption expenditure and trade levels rise, so will economic growth per capita in the MENA countries. Life expectancy (LLE) has a positive and statistically significant relationship with GDP per capita in the Panel OLS model, but it has a negative and insignificant relationship in the FMOLS and DOLS models.

## Discussion

 As the study aims to find a long-run relationship between the variables, the study first examines the stationarity of the data. Methodologically, when the series are integrated at first order, we can proceed with the test of cointegration. Based on the PP-Fisher chi-square test, the study finds that all variables are integrated at first order. As the study reports that the data are integrated at first order, cointegration is used to establish the long-run relationship between the variables. The study uses the Pedroni test and reports a cointegrating relationship between the variables. The result of this study confirms the finding of Lacheb et al^22^ and Mehrara et al,^24^ who also found a long-run cointegrating relationship between economic growth and health expenditure in the MENA region. Sethi et al,^12^ Piabuo and Tieguhong,^13^ Sahnoun,^15^ Rizvi,^16^ Sarpong et al,^17^ and Boussalem et al^26^ have all reported a similar cointegrating relationship between economic growth and health expenditure.

 Next, the study applies the Granger causality test to study the causal relationship between the variables. The presence of causality indicates that previous values of one variable are able to explain the current values of the other variable. The current study results indicate the absence of any causal relationship between health expenditure and economic growth. This result contradicts the findings of Piabuo and Tieguhong^13^ which reports bidirectional causality between health expenditure and economic growth for countries with lower health expenditure and unidirectional causality between health expenditure and economic growth for countries with higher health expenditure.

 The current study’s findings also contradict the findings of Sethi et al^12^ and Sarpong et al,^17^ who found bidirectional causality between economic growth and healthcare spending. The current study’s findings also contrast with those of Boussalem et al^26^ and Alhassan et al,^27^ who found unidirectional causality from health expenditure to economic growth; and Mehrara and Musai^23^ and Alhowaish,^30^ who found unidirectional causality from economic growth to health expenditure. The findings of this study, particularly about institutions, contradict the findings of Sethi et al,^12^ who found unidirectional causality running from institutions to health expenditure. Ghorbel and Kalai’s^29^ is the only study that reported similar results of no causality between healthcare and economic growth, as in the current study.

 Finally, the Panel OLS results show that institutions contribute negatively to growth. In contrast, the FMOLS and DOLS results show that institutions have a negligible contribution to economic growth. More importantly, there is no significant interaction between institutions and health expenditure. This result contradicts the findings of Sarpong et al,^17^ who found a significant interaction between health expenditure and institutional quality. This result also means that in the countries studied, institutions are not interacting positively so that healthcare boosts economic growth. Mehrara et al,^24^ Churchill et al,^28^ and Ghorbel and Kalai^29^ have all previously reported a negative impact of healthcare spending on economic growth. However, studies such as Sethi et al,^12^ Piabuo and Tieguhong,^13^ Sahnoun,^15^ Rizvi,^16^ Sarpong et al,^17^ Lacheheb et al,^22^ and Boussalem et al^26^ contradict the findings of the current study.

 The inclusion of institutions in the relationship between health expenditure and economic growth is a significant contribution of this study. According to Sethi et al,^12^ this relationship between health expenditure, institutions, and economic growth is still understudied. Furthermore, the authors discover no such study, particularly concerning the MENA region. An earlier study on Southeast Asian and Pacific countries by Rizvi^16^ found that health investments lead to higher economic growth when institutions are better. Furthermore, in his research on SSA countries Sarpong et al^17^ found that economic growth increases only when institutions interact positively with health capital. However, according to the findings of this study, institutional quality has no positive impact on the relationship between health expenditure and per capita income in the countries under consideration.

 The findings of this study give the impression that healthcare spending is a financial burden on the economy. The lack of a causal relationship between the two adds to the validity of the discovery. Nonetheless, because the two variables have a cointegrating relationship, they tend to move together in the long-run. Further insights from panel regression methods show that institutions do not significantly contribute to the sample countries’ economic growth. Taking a cue from this, the researchers feel that improving the functioning of institutions can catalyse the relationship between health expenditure and economic growth.

## Conclusion

 The study reports a negative correlation between healthcare spending and economic growth. This finding casts doubt on the healthcare systems in the countries studied. It implies that increased healthcare spending alone will not guarantee high economic growth. Other factors such as institutional quality, increased household consumption expenditure, trade, and life expectancy must be in place to facilitate economic growth. These facilitating factors would ensure that healthcare spending positively impacts growth. The presence of a long-run equilibrium relationship between the variables backs up this claim.

 This negative relationship indicates that a significant portion of these health investments is unproductive. This may be because of healthcare spending on the elderly and children. They contribute little or nothing to economic growth as they do not form the workforce. Supporting this, the study found no causality between health expenditure and economic growth. Towards this, the study identifies a detailed accounting of health expenditure on the unproductive segment of the population as a future research topic. Finally, this study recommends enhancing institutional quality indicators to aid health expenditure in stimulating economic growth.

 Since the study does not cover the entire MENA region, its findings apply to only the region’s seven countries. Also, many observable and unobservable social, economic, and political elements could not be included in the study. Hence these findings may not apply to other nations or regions worldwide. Even though the survey consists of several control variables and a fixed effect for year and country, the results may be imprecise due to the shorter study period and possible endogenous and unobservable variables that the study may have overlooked in the empirical econometric strategy. The researchers believe that future research can include the entire MENA region or other countries with more comprehensive longitudinal data.

## Authors’ contributions

 BUK and MIH designed and conceptualised the study. MRK and MRT collected the data. BUK and MIH analysed the data and interpreted the results. BUK, MIH, MRT and MRK drafted the manuscript. All authors contributed to the interpretation of the results and critical revision of the manuscript for important intellectual content and approved the final version of the manuscript. Finally, all authors reviewed the final draft and approved publishing.

## Funding

 None.

## Ethical approval

 Not required.

## Competing interests

 None.

## Data sharing

 All the data used in this paper comes from the World Development Indicator database of World Bank, which is publicly available using the link http://data.worldbank.org/data-catalog/world-development-indicators

## Appendix 1

**Appendix 1:** Descriptive statistics

**Variable**	**Obs**	**Mean**	**Std. Dev.**	**Min**	**Max**
**Country 1**
GDP	18	17116.78	5854.749	8684.647	25243.36
HE	18	4.249141	0.840263	2.971004	5.998345
LE	18	73.74206	0.725867	72.561	74.874
HC	18	1.56E+11	7.81E+10	6.86E+10	2.84E+11
LF	18	9703658	2519585	6358134	1.38E+07
TR	18	77.89056	10.84701	61.86	96.1
INS	17	-2.05094	0.416286	-2.90032	-1.28494
Country 2
GDP	18	4635.8	1978.767	1670.009	7927.847
HE	18	6.138222	1.292943	4.735038	8.859507
LE	18	73.31122	2.002135	70.176	76.271
HC	18	1.56E+11	7.25E+10	5.45E+10	2.85E+11
LF	18	2.37E+07	2035901	1.92E+07	2.74E+07
TR	18	46.20333	4.274876	39.02	54.44
INS	17	-5.81601	0.949159	-7.39392	-4.58884
Country 3
GDP	18	15272.99	4871.676	8475.964	22139.64
HE	18	3.020174	0.641347	2.012227	4.328745
LE	18	75.08533	1.603945	72.126	77.393
HC	18	1.67E+10	7.79E+09	6.90E+09	2.94E+10
LF	18	1440293	625443.2	786494	2601786
TR	18	97.55222	15.16263	77.02	128.47
INS	17	6.085085	0.505079	5.210553	7.045288
Country 4
GDP	18	59241.78	19782.07	28517.27	85076.15
HE	18	2.349446	0.559585	1.599962	3.52964
LE	18	78.79933	0.81964	77.467	79.981
HC	18	1.84E+10	1.31E+10	2.70E+09	4.11E+10
LF	18	1138481	647402.2	339550	2046136
TR	18	93.67167	7.752752	80.14	115.75
INS	17	3.745505	0.513002	2.954333	4.90917
Country 5
GDP	18	3532.447	736.707	2211.827	4307.156
HE	18	5.975273	0.754828	5.048344	7.232457
LE	18	74.78461	0.947393	73.172	76.31
HC	18	2.44E+10	6.66E+09	1.30E+10	3.29E+10
LF	18	3661197	298753.7	3221210	4036987
TR	18	95.79889	9.069415	82.39	114.35
INS	17	1.545363	0.675057	0.554355	2.794327
Country 6
GDP	18	25621.33	6117.937	14388.35	35397.36
HE	18	6.086978	0.628592	5.308001	6.901281
LE	18	79.24989	0.859273	78.009	80.672
HC	18	1.33E+10	4.03E+09	6.18E+09	1.86E+10
LF	18	549545.9	53553.54	445184	611083
TR	18	121.0394	13.07488	102.79	146.4
INS	17	2.902857	0.793854	1.964489	4.345609
Country 7
GDP	18	28209.84	7583.515	18435.89	40541.86
HE	18	7.079995	0.147256	6.807063	7.407464
LE	18	80.96423	1.143109	78.95366	82.55122
HC	18	1.19E+11	4.31E+10	6.70E+10	1.93E+11
LF	18	3373460	407574.5	2768248	4024831
TR	18	69.58722	8.046768	57.21	81.84
INS	17	-0.96816	0.533833	-1.67494	-0.04985

Source: World Development Indicators, World Bank. Available online at:http://data.worldbank.org/data-catalog/world-development-indicators
